# Cross-Talk Between Endothelin-1 and Mineral Metabolism in Hemodialysis Patients: A Cross-Sectional Study

**DOI:** 10.5812/ircmj.18115

**Published:** 2014-06-05

**Authors:** Jamal Halaj Zadeh, Amir Ghorbanihaghjo, Nadereh Rashtchizadeh, Hassan Argani, Shahnam Valizadeh, Najat Halaj, Amir Mansour Vatankhah

**Affiliations:** 1 Biotechnology Research Center, Tabriz University of Medical Sciences, Tabriz, IR Iran; 2 Urology and Nephrology Research Center, Shahid Beheshti University of Medical Sciences, Tehran, IR Iran; 3 Drug Applied Research Center, Tabriz University of Medical Sciences, Tabriz, IR Iran

**Keywords:** Hemodialysis, Parathyroid Hormone, Endothelin-1

## Abstract

**Background:**

Endotheline-1 (ET-1), an endothelial mediator, influences on mineral metabolism; especially vascular calcification in uremic patients.

**Objectives:**

The aim of the present study was to evaluate of ET-1, high-sensitivity C-reactive protein (hs-CRP) and mineral metabolites as the main factors for vascular calcification and inflammation in hemodialysis (HD) patients.

**Patients and Methods:**

In this cross-sectional study, 46 chronic stable HD patients were selected from nephrology departments of Tabriz University of Medical Sciences affiliated hospitals and classified based on phosphorus (P), Ca-P product (Ca × P) and intact Parathyroid Hormone (iPTH) levels. We evaluated fasting serum ET-1and hs-CRP levels by the standard methods and compared with 46 healthy control subjects.

**Results:**

The levels of serum hs-CRP and ET-1 were significantly higher in the patient’s group compared with controls (4.40 ± 1.26 vs. 1.38 ± 1.61, P < 0.0001, and 2.31 ± 0.87 vs. 0.75 ± 0.48, P < 0.0001, respectively) and with regard to Ca × P product cut-off point (3.99 ± 0.78 vs. 5.33 ± 1.64, P < 0.0001, and 2 ± 0.73 vs. 3.04 ± 0.73, P < 0.0001 respectively). ET-1 was correlated significantly with hs-CRP level (r = 0.776, P < 0.0001). Serum P, Ca × P and iPTH levels directly and Ca indirectly were correlated with serum ET-1 in HD patients (r = 0.932, P < 0.0001, r = 0.766, P < 0.0001, r = 0.514, P < 0.0001, r = -0.538, P < 0.0001 respectively). Multiple regression analysis demonstrated that serum P were independently associated with ET-1 levels (β = 0.932, P < 0.0001).

**Conclusions:**

Serum P and iPTH levels were independently associated with ET-1 and those may play a role in development of endothelial dysfunction in chronic kidney disease.

## 1. Background

Cardiovascular events rate are markedly increasing in hemodialysis (HD) patients. Approximately 50% deaths of patients with end stage renal disease (ESRD) originate from cardiovascular diseases (1). Cardiovascular mortality in ESRD population is 10 to 30 times more than general population (2). It has been shown that endothelial dysfunction (ED) is one of the major risk factors for development of atherosclerosis and subsequent cardiovascular events in HD patients (3, 4).

Endothelins (ETs) are a family of 21 amino acid peptides including Endothelin-1 (ET-1), Endothelin-2 (ET-2), Endothelin-3 (ET-3) and Endothelin-4 (ET-4); each with distinct genes and tissue distributions. ET-1 is the major endothelial isoform that originally isolated from porcine aortic endothelial cells (5-7). ET-1is a pleiotropic molecule, best known for its action as a potent constrictor (8) and vascular endothelium is an abundant source of ET-1 (9, 10). ET-1 gene transcription is enhanced with various stimulants such as pro-inﬂammatory cytokines, hyperglycemia, acidosis and thrombin. Inﬂammation is the other important factor for atherosclerosis process in HD patients. The prognostic value of high sensitivity C-reactive protein (hs-CRP) in prediction of cardiovascular disease has been suggested by many investigators. Elevation of hs-CRP is strongly associated with vascular disease. It has been suggested that this protein not only is a marker, but also acts as a mediator in atherogenesis. In ESRD, hs-CRP has been proven to be associated with endothelial dysfunction. Pasceri et al. (11) showed that hs-CRP in endothelial cell of umbilical vein culture, stimulates endothelin-1 releasing, a potent endothelial-derived contracting factor. Despite of high prevalence and relevance of alterations in mineral metabolism in the chronic kidney diseases (CKDs), those relationships with inflammation and ET-1 have scarcely been explained.

## 2. Objctives

Our aim from this study was to evaluate the correlation of hs-CRP and ET-1, as markers of endothelial dysfunction, and mineral metabolites in the HD patients.

## 3. Patients and Methods

### 3.1. Participants

The study was performed in the Biochemistry and Hemodialysis Department of Tabriz University of Medical Sciences (TUOMS) affiliated hospitals, Iran. The ethics committee of TUOMS approved the study and informed consent was obtained from all the participants in the study. Recruitment of the patients occurred from March 2011 to October 2011. Forty six (28 males and 18 females) stable HD patients without any active infection, malignancy, viral hepatitis, or chronic inﬂammatory diseases and 46 age and race matched Healthy Controls (HC) (23 males and 23 females) were included in the study. Sample size was calculated by sample size formula based on ET-1 concentration (α = %5, β = %10). Patients treated by Parathormone or history of cardio vascular diseases were excluded from the study. The causes of renal failure in the patients were diabetic nephropathy (41.3%), chronic glomerulonephritis (8.6%), polycystic kidney disease (10.8%), hypertensive ischemic nephropathy (19.5%), obstructive nephropathy (15.2%) and unknown etiology (4.3%). All of the HD patients were under regular hemodialysis for at least six months (6-84 months) in three sessions of 4 hour per week by synthetic high-ﬂux membranes with Fresenius-2008B hemodialyser.

### 3.2. Laboratory Analyses

Blood samples for biochemical evaluations were drawn prior to a dialysis session (after 12 hours of overnight fasting). Serum samples were separated within 30 minutes and stored at -80ᵒC until tests were done. The following parameters were measured: serum total calcium (Ca), phosphorus (P), alkaline phosphatase (ALP), intact PTH (iPTH), albumin (Alb), cholesterol (Ch), triglycerides (TG), urea, creatinine (Cr) and hs-CRP. Biochemical parameters were measured by colorimetric methods with an automated chemical analyzer (Abbott analyzer, Abbott laboratories, Abbott Park, North Chicago, IL). Total calcium was corrected for serum albumin by this equation: Calcium = Ca + 0.8 [4.0-albumin (g/dL)]. iPTH level was measured by two-site ELISA method [Enzyme-Linked Immunosorbent Assay] (Immunodiagnostic System, Bolden, UK), with the sensitivity of 1.57 pg/mL. ET-1 serum concentration was determined with a Chemiluminescent Immunoassay method for human Endothelin (QuantiGlo® ELISA kit, Reutlingen, Germany), which uses a high affinity monoclonal antibody specific for this protein. The minimum detectable dose (MDD) with inter-assay coefﬁcient of variation (CV) 9.1% and intra-assay CV of 3.4% was 0.064 pg/mL hs-CRP concentration was measured by nephelometric method (Pars Azmoon Co).

### 3.3. Statistical Analysis

All data were analyzed by Kolmogorov-Smirnov test for normality of distribution. Results are expressed as mean ± SD for parametric data and as median (inter quartile range) for nonparametric data. Numbers (%) are shown when appropriate. Differences among groups were assessed by Mann-Whitney-U test for the nonparametric data or by independent samples t-test for parametric data. We further determined correlations between all variables with Pearson’s correlation test. The null hypothesis was rejected at a two-tailed P < 0.05. Differences between groups were evaluated using ANOVA test. Simple and multiple regression analyses were used to test the relationships between the variables as appropriated. Statistical analysis was performed using SPSS version 18.

## 4. Results

Characteristics of HD patients and HC are shown in Table 1. There were no signiﬁcant differences in the mean age and sex between the two study groups. Up to 69.56% (n = 32) of patients had serum P above the upper limit of 5.5 mg/dL (12) and the 34.78% of patients (n = 16) had Ca × P product above the upper limit of 55 mg/d (12, 13).

The mean iPTH concentration was 367.29 ± 133.38 pg/mL; in 69.56% of patients (n = 32) iPTH was above the upper limit of 300 pg/mL (12). As shown in Table 1, concentration is higher in the HD than the control group, [67.5%, P < 0.0001]. Table 2 shows that serum levels of ET-1 did not differ between males and females in the HD and control groups [P = 0.1, P = 0.43 respectively]. A signiﬁcant difference in serum hs-CRP concentration between the HD and HC was found; Serum hs-CRP concentration in the HD patients was higher than the control group [61.8%, P < 0.0001]. Serum hs-CRP concentration was almost equal in the males and females in the HD [P = 0.08] and HC [P = 0.43] groups (Table 2). After dividing the patients group into two subgroups according to the Ca × P product cut-off point (12) (< 55 and > 55 mg/dL, respectively), hs-CRP and ET-1 were found to be signiﬁcantly different between the two subgroups (Table 1). Further analysis was conducted for evaluating the patients group according to Ca × P product, P and iPTH (Table 3). Plasma levels of hs-CRP and ET-1 showed a trend to increase that led to a signiﬁcant difference between tertile subgroups. hs-CRP and ET-1 values reached statistical signiﬁcance between Ca × P product tertiles (ANOVA; P < 0.0001, P < 0.0001, respectively). Regarding P, serum hs-CRP and ET-1 levels were also higher in patients with highest tertile (ANOVA; P < 0.0001, P < 0.0001, respectively). Higher concentrations of hs-CRP and ET-1 were shown in patients with the highest tertile of iPTH (ANOVA; P < 0.04, P < 0.02, respectively).

Table 4 shows potential correlations from the bivariate analysis examining ET-1 and serum P, Ca × P and iPTH. The independent relationship of mineral metabolism and inflammation was evaluated. Multiple regression analysis showed that among different independent variables, only serum P was independently associated with ET-1 (β = 0.932 P < 0.0001) (Table 5).

**Table 1.** Demographic and Clinical Characteristics of Reference Controls, of 46 End-Stage Renal Disease (ESRD) Patients on Hemodialysis Treatment, and of the Two Subgroups Obtained According to Ca × P Product Cut-off point ^a,b^

Variable	HD Group (n = 46)	HC Group (n = 46)	P Value^c^	Ca × P ≤ 55, mg/dL (n = 32)	Ca × P > 55, mg/dL (n = 14)	P Value ^d^
**Age, y**	61.08 ± 13.92	61.84 ± 1152	NS	61.46 ± 15.32	60.21 ± 10.47	NS
**Sex, male/female**	28/18	23/23	-	22/10	6/8	-
**SYS, mmHg**	141.8 ± 20.6	108.9 ± 12.9	0.000	140.1 ± 19.8	143.9 ± 21.2	NS
**DIA, mmHg**	77.3 ± 10.2	75.4 ± 9.6	NS	78.6 ± 9.6	76 ± 10.8	NS
**Time on dialysis, month**	44 ± 34.4	-	-	45.7 ± 33.3	43 ± 30	NS
**Alkaline phosphatase, IU/L**	411.15 ± 310.05	185.76 ± 59.93	0.000	459.12 ± 354.65	301.50 ± 119.26	0.0001
**Calcium, mg/dL**	8.81 ± 0.90	9.50 ± 0.56	0.000	8.67 ± 0.56	9.46 ± 1.31	0.0001
**Ca × P product, mg/dL**	53.43 ± 9.74	38.73 ± 7.82	0.000	48.66 ± 5.86	64.34 ± 7.88	0.0001
**Albumin, g/dL**	3.48 ± 0.77	3.98 ± 0.49	0.000	3.50 ± 0.78	3.45 ± 0.78	NS
**Total protein, g/dL**	8.25 ± 1.01	7.88 ± 1.3	NS	8.16 ± 1.11	8.47 ± 0.72	NS
**Phosphorus, mg/dL**	6.05 ± 0.91	4.08 ± 0.87	0.000	5.71 ± 0.71	6.85 ± 0.82	0.0001
**iPTH, pg/dL**	367.29 ± 133.38	26.04 ± 15.34	0.000	157.46 ± 179.28	397.91 ± 151.38	0.0001
**Creatinine, mg/dL**	9.20 ± 2.44	1.10 ± 0.28	0.000	9.14 ± 2.2	9.35 ± 2.91	NS
**Urea, mg/dL**	104 ± 17.47	39.56 ± 16.05	0.000	99.71 ± 14.43	114 ± 20.18	0.0001
**Cholesterol, mmol/L**	82.88 ± 14.41	79.1 ± 44.29	NS	82.88 ± 14.59	83.42 ± 15.5	NS
**Triglyceride, mmol/L**	169.1 ± 53.9	97.4 ± 35.6	0.000	165 ± 56	167 ± 50	NS
**hs-CRP, mg/L**	4.40 ± 1.26	1.38 ± 1.61	0.000	3.99 ± 0.78	5.33 ± 1.64	0.0001
**Glucose, mg/dL**	126 ± 7.21	92.43 ± 28,83	NS	132.3 ± 74.1	111.8 ± 46.98	NS
**ET-l, pg/mL**	2.31 ± 0.87	0.75 ± 0.48	0.000	2 ± 0.73	3.04 ± 0.73	0.0001

^a^ Abbreviations: DIA, diastolic; ET-1, endothelin-1; HC, health control; hs-CRP, high sensitive C reactive protein; HD, hemodialysis patients; iPTH, intact parathormone; NS, non-significant; SYS, systolic.

^b^ Data are presented as Mean ± SD.

^c^ P < 0.05 vs. controls.

^d^ P < 0.05 subgroup Ca × P, 55 mg/dL vs. subgroup Ca × P > 55 mg/dL.

**Table 2.** Comparison of the Serum Levels of ET-1and hs-CRP Between Males and Females in Two HD and HC Study Groups ^a,b^

Parameters	Male	Female	P Value^c^
**ET-1, ng/mL**			
HD	2.15 ± 0.73 (58.69)	2.57 ± 1.02 (41.3)	0.1
HC	0.8 ± 0.6 (50)	0.69 ± 0.34 (50)	0.43
P value^d^	0.0001	0.0001	-
**hs-CRP, ng/mL**			
HD	4.14 ± 0.83 (58.69)	4.79 ± 1.69 (41.3)	0.08
HC	1.2 ± 1.42 (50)	1.57 ± 1.78 (50)	0.43
P value^e^	0.0001	0.0001	-

^a^ Abbreviations: HD, hemodialysis patients; HC, health control.

^b^ Data are presented as Mean ± SD, (%).

^c^ Differences among groups in ET-1 and hs-CRP were assessed by Independent-Sample t-test, Serum ET-1 and hs-CRP levels (males vs. females).

^d^ Serum ET-1 levels (HD group vs. HC group).

^e^ Serum hs-CRP levels (HD group vs. HC group).

**Table 3.** Plasma Concentrations of ET-1, iPTH and hs-CRP According to the Tertiles of Ca × P Product, P and iPTH in Hemodialysis Patients ^a^

	Tertile I, n = 14	Tertile II, n = 17	Tertile III, n = 15	P Value ^b^
**Tertiles of Ca × P product**				
ET-l, pg/mL	1.42 ± 0.39	2.41 ± 0.58	3.04 ± 0.73	0.0001
hs-CRP, mg/L	3.3 ± 0.36	4.51 ± 0.56	5.29 ± 0.73	0.0001
**Tertiles of phosphorus**	n = 16	n = 13	n = 17	
ET-l, pg/mL	1.4 ± 0. 35	2.51 ± 0.42	3.03 ± 0.69	0.0001
hs-CRP, mg/L	3.46 ± 0.7	4.37 ± 0.36	5.31 ± 1.47	0.0001
**Tertiles of iPTH**	n = 15	n = 16	n = 15	
ET-l, pg/mL	1.88 ± 0.73	2.30 ± 0.59	2.76 ± 1.05	0.02
hs-CRP, mg/L	3.98 ± 0.92	4.18 ± 0.63	5.04 ± 1.78	0.04

^a^ Abbreviations: iPTH, intact parathormone; ET-1, endothelin-1; hs-CRP, high sensitive C reactive protein.

^b^ ANOVA test.

**Table 4.** Correlation Coefficients (r) of ET-1 With hs-CRP, Ca and P in Hemodialysis Patients^a^

Variables	ET-1	P
**Calcium, mg/dL**	-0.538	0.0001
**Phosphorus, mg/dL**	0.932	0.0001
**Ca × P, mg/dL**	0.766	0.0001
**iPTH, pg/dL**	0.514	0.0001
**hs-CRP, mg/L**	0.776	0.0001

^a^ Abbreviations: iPTH, intact parathormone; ET-1, endothelin-1; hs-CRP, high sensitive C reactive protein.

**Table 5.** Multiple Regression Analysis for ET-l as Dependent Variable^a^

Independent Variable	Beta-regression Coefficient	SE of Regression Coefficient	t	P Value
**hs-CRP, mg/L**	-0.068	0.083	-0.565	0.575
**Ipth, pg/dL**	0.033	0.001	-0.414	0.681
**Calcium, mg/dL**	-0.076	0.140	-0.901	0.373
**Phosphorus, mg/dL**	0.932	0.158	5.650	0.0001
**Ca ×P, mg/dL**	0.006	0.009	0.054	0.957
**Vitamin D 25, OH**	-0.042	0.009	-0.585	0.562
**ALP, IU/L**	0.003	0.000	0.052	0 .959

^a^ Abbreviations: ALP; Alkaline phosphatase, ET-1; Endothelin-1, hs-CRP; high sensitive C, iPTH; intact Parathormone, reactive protein.

## 5. Discussion

There is a strong relationship between elevated serum P, Ca × P product, and parathyroid hormone and cardiac causes of death in HD patients, especially deaths resulting from CAD and sudden death (13). In this study, we explored the association of mineral markers of metabolism (Ca, P, and PTH) and inflammation parameters (hs-CRP) with endothelin-1 in hemodialysis patients. We observed significant differences in plasma ET-1 levels in the two subgroups obtained by dividing the whole group according to Ca × P product cut-off point. These data indicate relationship between ET-1 and mineral metabolism in HD patients. A further analysis conducted after dividing the overall group according to Ca × P product, P and iPTH showed a direct correlation with increase of ET-1 concentration. Chang et al. (14) reported that ET-1 increased PTH release in a dose-dependent manner in human parathyroid cells. A recent study demonstrated that the ET-1 receptor blocker, Bosentan, failed to reduce parathyroid hyperplasia and PTH values in 5/6 of nephrectomized rats. In contrast, Palermo et al. showed an inverse and independent relationship between the circulating levels of PTH and ET-1 in HD patients. The results of the present study seem to be against with results of Palermo et al. (15) bivariate correlation analysis showed that PTH is directly correlated with ET-1. Such a correlation seems to be logic as high levels of both ET-1 and PTH are usually found in CKD patients, and both could participate in enhancing CV damage in this population.

Although numerous observational studies have demonstrated association between CKD and excess cardiovascular risk (CV) (16-18), the underlying pathophysiological mechanisms of this relationship still need more investigation. In CKD, there are often clusters of classical CV risk factors such as hypertension and diabetes mellitus. It should be noted that in these patients, particularly in late-stage CKD, the role of classical risk factors in the prediction of CV outcomes are not well identified as in the general population. Indeed, in individuals with established renal failure (ERF) there is an inverse relationship between CV event rates and conventional risk factors such as total cholesterol, obesity and even blood pressure (19). These paradoxical associations may, at least in part, be explained by “reverse causality”, a feature of chronic disease malnutrition-inﬂammation syndromes (20). The failure of conventional risk factors to fully account for the excess CV mortality seen in CKD has fuelled ongoing interest into a number of emerging risk factors, most notably endothelial dysfunction.

Many risk factors, traditional and non-traditional, are thought to have major or minor role in development and progression of CVD in CKD patients. Some of these are established cardiovascular risk factors, for example: hypertension and smoking and their successful treatment or cessation results in reducing cardiovascular events and slowing down its progression. Others just seem to identify patients who are amongst at risk, such as high serum homocysteine level. Traditional and nontraditional risk factors for atherosclerosis is further complicated in patients with progressive CKD, because of the appearance of uremia-speciﬁc risk factors with the potential of contributing to endothelial and vascular dysfunction and damage.

Many novel putative ‘biomarkers’ of risk, either for CVD or for progression, have been discovered in the last two decades. For many of them causality has not been proven yet, even in experimental studies, and for almost all of them the deﬁnitive conﬁrmation of their pathophysiological role and clinical relevance from intervention trials in CKD patients is still pending.

In accordance with the findings in the general population, several studies have shown that elevated CRP predicts both all cause and cardiovascular mortality in hemodialysis (HD) (21-23). A significant difference of hs-CRP has been observed between HD subjects and healthy controls in our study. Our results are in accordance with the previous studied. Although markedly elevated plasma concentrations of proinflammatory cytokines, such as IL-6, have been documented in most ESRD patients, the origin of inflammation in patients with chronic renal disease remains unclear. However, it seems that both non-dialysis-related factors, such as reduction of kidney function per seconds, (24, 25) clotted access graft (26), failed kidney grafts (27), atherosclerosis (28) and persistent infections (29-31), and dialysis-related factors, such as generation of complement fractions as a result of plasma protein-membrane contact, the back filtration of contaminated dialysate to the blood compartment, and the direct contact of blood cells with the dialysis membrane (31, 32) might contribute.

Chronic inﬂammation results in endothelial dysfunction by quenching the production of NO and diminishing its bioactivity, (33) and facilitates the interactions between modiﬁed lipoproteins, monocyte-derived macrophages (34). Studies, largely in endothelial and monocyte-macrophage and vascular smooth muscle cells, support the role for hs-CRP in atherogenesis. In the venous endothelium, hsCRP has been shown to promote the release of the potent endothelial-derived contracting factor ET-1 (32). ET-1 not only is a potent vasoconstrictor but also appears to be a mediator of hs-CRP-induced up-regulation of adhesion molecules and monocyte chemo-attractant protein-1 in venous EC. Accumulating evidence suggests that high concentrations of hs-CRP, directly effects on endothelial cells dysfunction. Verma et al. (35) in vitro study confirmed that pro-atherogenic effects of hs-CRP were potentiated in the presence of hyperglycemia with increasing of ET-1 production and up regulated adhesion molecules and monocyte chemoattractant chemokine (MCP-1) expression in venous endothelial. There is insufficient data on the correlation between ET-1 and hs-CRP in hemodialysis patients up to now. Our study showed that correlation of hs-CRP with ET-1 is significant, both in the HC and HD groups, although it is more significant in the later (r = 0.366, P = 0.012, r = 0.835 P < 0.0001, respectively). It appears that uremic situation have a reinforcement effect on correlation between hs-CRP and ET-1. liu et al. in HD patients, found a close correlation between serum ET-1 level and hs-CRP (P < 0.01) and showed that serum ET-1 level, but not the hs-CRP, was signiﬁcantly associated with the extent of carotid atherosclerosis (36).

Plasma levels of hs-CRP showed a trend to increase with higher of P, Ca × P and iPTH concentration. Our data indicate a relation between ET-1 and mineral metabolism in HD patients in according with study of Palermo et al. In further analysis, which was conducted after dividing the HD group according to tertiles of iPTH, P and Ca × P, showed a trend toward a progressive increase of ET-1 concentration in the higher tertiles. The results of the present study indicate that elevated serum P is an independent predictor for increased levels of endothelial dysfunction in these patients, suggesting that hyperphosphatemia may promote and/or facilitate the development of endothelial disorder in CKD patients. The mechanisms which high phosphate levels adversely affect cardiovascular function are poorly understood. In Vitro, studies have shown that supraphysiological concentrations of phosphate reduce eNOS expression and no production in HUVECs (37). The simulated hyperphosphatemia induces down-regulation of eNOS expression that could be reversed by co-treatment with PFA, which is a specific inhibitor of phosphate transport across the cell membrane (37).

On the other hand, Previous studies support the role of elevated levels of ET-1 paralleling a decrease in eNOS expression, including acute renal failure (38), hypercholestrolemia and atherosclerosis (39). Therefore, it is possible to speculate that increased P concentration may trigger ET-1 releasing-driven signaling endothelial dysfunction cascades.

Measuring only few markers in ESRD patients in our study is one of our main limitations; however, the obtained results may be the basis for further studies on many other factors, which may be involved in the pathogenesis of this disease.

In conclusion, the present study demonstrated that ET-1 have positive correlation with PTH in the ESRD patients undergoing HD. This result seems to be inconsistent with the report that whether ET-1 with well-known harmful actions on CV is also able to inhibit PTH secretion. This is the first study that investigates the potential association between mineral metabolites and ET-1 in the HD patients, showing that serum P is a risk factor for the presence of an endothelial dysfunction accompanied with increased ET-1 level. Our observation on the association between serum P and ET-1 requires further exploration and confirmation by longitudinal prospective studies.
